# Comparing intramuscular progesterone, vaginal progesterone and 17 -hydroxyprogestrone caproate in IVF and ICSI cycle

**Published:** 2011

**Authors:** Ashraf Moini, Fatemeh Zafarani, Bita Eslami, Maria Sadeghi, Zahra Kamyabi, Nadia Jahangiri

**Affiliations:** 1 Department of Endocrinology and Female Infertility, Royan Institute of Reproductive Biomedicine, ACECR, Tehran, Iran.; 2 Department of Obstetrics and Gynecology, Arash Women’s Hospital, Tehran University of Medical Sciences, Tehran, Iran.; 3 Department of Reproductive Imaging, Royan Institute, ACECR, Tehran, Iran.

**Keywords:** *Invitrofertilization*, *Lutealphase*, *Progesterone*, *Pregnancy*

## Abstract

**Background:** Supplementation of luteal phase with progesterone is prescribed for women undergoing routine IVF treatment.

**Objective:** The objective of this study was to compare the efficacy of three types of progesterone on biochemical, clinical and ongoing pregnancy rates and abortion and live birth rates.

**Materials and Methods:** A prospective randomized study was performed at Royan Institute between March 2008 and March 2009 in women under 40 years old, who use GnRH analog down-regulation. One hundred eighty six patients in three groups were received progesterone in oil (100 mg, IM daily), intravaginal progesterone (400 mg, twice daily) and 17-α hydroxyprogestrone caproate (375mg, every three days), respectively.

**Results: **Final statistical analysis after withdrawal of some patients was performed in 50, 50 and 53 patients in group 1, 2 and 3 respectively. No differences between the groups were found in baseline characteristics. No statistical significance different was discovered for biochemical, clinical and ongoing pregnancies. Although the abortion rate was statistically higher in group 1 (p=0.025) the live birth rate was not statistically significant between the three groups.

**Conclusion: **The effects of three types of progesterone were similar on pregnancies rate. We suggest the use of intravaginal progesterone during the luteal phase in patients undergoing an IVF-ET program because of the low numbers of abortions, and high ongoing pregnancy rates.

## Introduction

In order to establish a successful pregnancy complex preparation is necessary. Supplementation of the luteal phase with progesterone is prescribed for women undergoing routine IVF treatment. Late luteal phase hormonal deficiencies may impair endometrial growth possibly leading to failure or abnormal implantation (1).

Previous studies have demonstrated the importance of progesterone administration in order to support the luteal phase (2). The most common route of administration in progesterone supplementation is via intramuscular (IM) oil injection, which can sometimes lead to severe inflammation, pain and sterile abscesses. Another route of administration is the vaginal insertion of suppositories which are easier to tolerate, however the suppository material may escape from the vagina thus leading to inconvenience and uncertainty as to the absorbed dosage of progesterone. 

Some studies have shown that IM progesterone has significantly higher embryo implantation, clinical pregnancies and live birth rates in comparison with Crinone 8% vaginal progesterone gel (3, 4). study by Chantilis *et al* has shown that for all age categories, positive beta-hCG and ongoing pregnancy rates were similar when either Crinone or IM progesterone were given for luteal phase support (5).

The compound pharmacokinetics of 17- hydroxyprogestrone caproate (17-HPC) are different and should only be administered every three days, thus reducing the total number of injections needed. A previous report has confirmed a higher pregnancy rate in the group supported by 17-HPC than those who received placebo following embryo transfer (ET) (6). A comparison of IM progesterone and 17-HPC for biochemical, clinical, and ongoing pregnancies has revealed no statistically significant differences (7). 

The objective of this study was to compare the efficacy of three types of progesterone on biochemical, clinical and ongoing pregnancy rates. The present study has also considered the abortion and live birth rates. 

## Materials and methods

A prospective randomized study was performed in all patients who visited the IVF Unit of Royan Institute between March 2008 and March 2009. The Institutional Review Board of Royan Institute approved the protocol and informed consent was obtained from all participants. The inclusion criteria were the use of GnRH analog down-regulation and less than 40 years of age.

In all patients, administration of GnRH-a (0.5mg/day, Suprefact, Hoechst, Frankfurt, Germany) began on day 21 of their menstrual cycles and continued until the second day of the next menstrual cycle. Subsequently the dosage of GnRH-a was decreased to 0.2mg/day. Ovarian hyperstimulation was defined as serum 17-β Estradiol level≥3500 Pg/ml. Patients were monitored by the size and number of follicles, and endometrial thicknesses on days 5, 7 and 12 of stimulation. All patients underwent transvaginal ultrasound which was performed by one sonographer. After observation of at least three follicles with diameter(s) that exceeded 18 mm, 10000 IU of hCG (IM) was injected. Oocytes were retrieved 34-36 hours following hCG administration by transvaginal echoguided aspiration. The IVF medium (Vitrolife) was used as culture medium. Spermatozoa were prepared with the swim-up technique. ET was performed at the two to four cell stages, 40-44 hours after insemination. No greater than three embryos were transferred. The quality of embryos transferred was similar in all groups. Patients were assigned to receive one of the three treatments by a computer-generated randomization schedule, which started from the evening of oocyte retrieval. Patients in group 1 were administered progesterone in oil (100mg, IM daily, Pars Minoo, Iran), those in group 2 were given intravaginal progesterone (400mg, twice daily, Cyclogest, Chemist Direct, UK) and group 3 received 17HPC (375mg, every three days, Pharmax Co., Turkey). Treatment continued until pregnancy test results. Enrollment and assignment of patients was performed by one researcher. Owing to differences between the drugs, it was not possible to blind participants.

The study participants were evaluated for biochemical, clinical and ongoing pregnancies. Abortion and live birth rates were also studied. 

A biochemical pregnancy was defined as a small increase in β-hCG levels, whereas a clinical pregnancy was defined by the visualization of an embryo with cardiac activity at 6-7 weeks of pregnancy. The live birth rate was clarified as the number of live birth deliveries per 100 ET cycles. Ongoing pregnancy rate was defined as a viable pregnancy with≥20 weeks of gestation. The clinical abortion rate was estimated as the proportion of spontaneous clinical abortions to the total number of clinical pregnancies.

Sample size calculation was based on a study by Damario *et al* (4) which determined the biochemical pregnancy rate (percent of positive hCG titers) to be approximately 30% in Crinone, and 10% in IM progesterone. On the basis of this estimate, 62 patients were required in each group in order to detect differences with a 5% level of significance and 80% power.

**Statistical analysis**

Statistical analysis was performed with SPSS software (version 13). Statistical significance for the differences was tested by χ^2^-test, ANOVA and the Kruskal-Wallis test, when appropriate. A p-value less than 0.05 was considered statistically significant.

## Results

**Figure 1:**
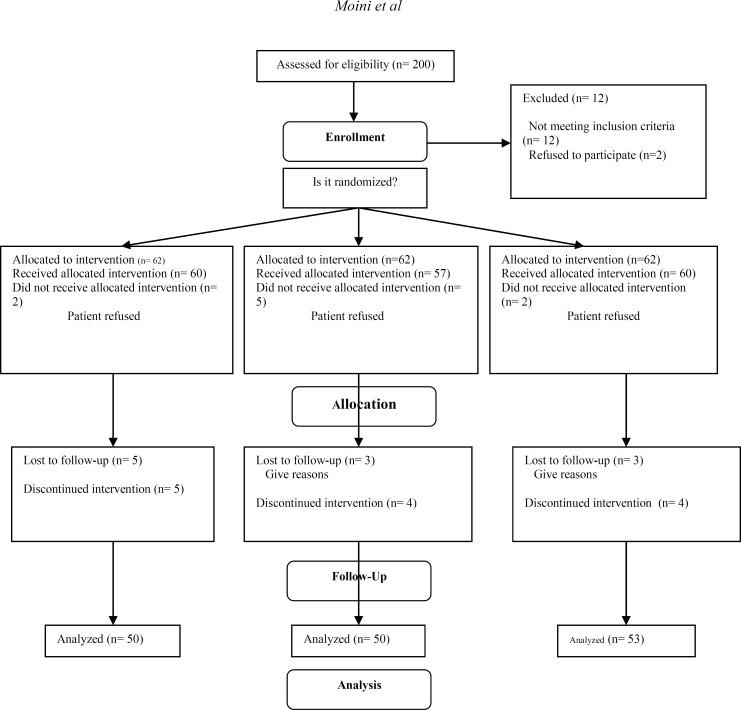
Flowcharts of patient participations

There were 186 patients who met the inclusion criteria and were randomly assigned to three groups. Some patients withdrew consent from the study (Figure 1), therefore for analysis; there were 50, 50 and 53 patients in groups 1, 2 and 3 respectively who continued participation. 

No differences between the groups were found in terms of mean age, body mass index, etiology and duration of infertility, presence of primary/ secondary infertility, menstrual pattern and type of cycle (Table I). Meanwhile, the endometrial thicknesses on the ET day was similar between the three groups (9.35±1.41, 9.29±1.53, and 9.23±1.55 mm: ANOVA test= 0.92). 

Based on Table II, no statistical significance was discovered for biochemical, clinical and ongoing pregnancies in the three groups (p>0.05). However the abortion rate was statistically higher in group 1 when compared with the other groups (35.3%, 5.9%, 5.9%: p= 0.025). Meanwhile the live birth rate was not statistically significant between the three groups.

**Table I:** Baseline patient characteristics

	**Group 1**	**Group 2**	**Group 3**	**p-value**
	**(n=50)**	**(n=50)**	**(n=53)**	
Mean age, years (SD)	32.71 ± 4.03	32.19 ± 4.73	32.13 ± 4.27	0.76
Mean BMI, kg/m2 (SD)	25.08 ± 3.64	25.79 ± 4.29	26.27 ± 4.50	0.42
Endometrial thickness (mm)	9.35 ± 1.41	9.29 ± 1.53	9.23 ± 1.55	0.92
Infertility type				
- Primary	46 (90.2)	44 (84.6)	50 (94.3)	
- Secondary	5 (9.8)	8 (15.4)	3 (5.7)	0.26
Menstrual pattern				
- Irregular	16 (31.4)	11 (21.2)	9 (17)	
- Regular	35 (68.6)	41 (78.8)	44 (83)	0.20
Infertility factor				
- Anovulation	17 (33.3)	14 (26.9)	14 (26.4)	
- Endometriosis	2 (3.9)	1 (1.9)	0 (0)	
- Male factor	16 (31.4)	26 (50)	29 (54.7)	
- Tubal factor	6 (11.6)	7 (13.5)	3 (5.7)	
- Other	9 (17.6)	4 (7.7)	7 (13.2)	0.30
Type of cycle				
- IVF	7 (13.7)	5 (9.6)	3 (5.7)	
- ICSI	27 (52.9)	32 (61.5)	28 (52.8)	
- IVF/ICSI	17 (33.3)	15 (28.8)	22 (41.5)	0.48

BMI: Body Mass Index; SD: Standard Deviation.

Group 1: progesterone in oil;

Group 2: intra-vaginal progesterone

Group 3: 17- hydroxyprogestrone caproate

**Table II:** Pregnancy outcomes of patients in the three study groups

	**Group 1**	**Group 2**	**Group 3**	**p- value**
	**(n=50)**	**(n=50)**	**(n=53)**	
Biochemical pregnancy	38% (19/50)	38% (19/50)	28.3% (15/53)	0.49
Clinical pregnancy	34% (17/50)	34% (17/50)	26.4% (14/53)	0.63
Abortion rate	35.5% (6/17)	5.9% (1/17)	7.1% (1/14)	0.025
Ongoing pregnancy	20% (10/50)	32% (16/50)	24.5% (13/53)	0.27
Live birth rate	18% (9/50)	32% (16/50)	24.5% (13/53)	0.38

## Discussion

This study demonstrated that biochemical and clinical pregnancy rates were similar between progesterone IM and the intravaginal group, whereas they were lower in the 17-OHP group (not statistically significant). 

On the other hand, according to the results, the ongoing pregnancy and live birth rates were higher in the intravaginal group when compared with the other groups. However the differences were not statistically significant. A retrospective cohort study indicated the first trimester progesterone supplementation may support early pregnancy through 7 weeks by delaying miscarriage but does not improve live birth rates (8). 

Clinical outcomes in the Damario *et al* study revealed that the use of Crinone 8% vaginal progesterone gel was associated with an increased incidence of biochemical pregnancies (4). However IM progesterone had significantly higher clinical pregnancy, embryo implantation and live birth rates when compared with Crinone 8% vaginal progesterone gel (4). 

The result of our study differed from the Damario investigation. However, the conclusion of another study by Chantilis *et al* has shown that positive beta-hCG results were similar when Crinone or IM progesterone was given for luteal phase support (5) which confirmed the present study results. Two recent studies which compared the efficacy of intravaginal progesterone gel and intramuscular progesterone showed the similar outcomes in pregnancy, whereas fewer side effects and greater overall satisfaction were reported by women receiving Crinone (9, 10). On the basis of some studies, the use of 17-HPC was emphasized for luteal phase support following IVF when compared with placebo (6).

The similarity of the results for biochemical, clinical, and ongoing pregnancy rates between two groups who received 17-HPC and IM progesterone in the Costabile *et al* study encouraged the use of 17-HPC for luteal phase support (7). 

In addition another study by Abu-Musa *et al* was conducted to assess the effect of 17-HPC on the pregnancy outcome on IVF-ET cycles revealed no significant difference in the pregnancy rate between cases (17-HPC group) and control (no injection) (11). Our study support the hypothesis that 17-HPC can replace IM progesterone due to the higher rates of ongoing pregnancies and lower number of abortions. 

The high number of abortion in IM progesterone group maybe due to the fact that IM progesterone in oil generate circulating progesterone concentrations at or above the physiological range and vaginally administered progesterone yield lower serum level, but nonetheless achieved endometrial tissue concentrations up to 30-fold greater than those achieved with IM progesterone (12). 

Moreover, a study which compared three groups (group I intramuscular progesterone, group II vaginal progesterone and group III unsupported) revealed statistically significant pregnancy rates only between group I and groups II and III, however between groups II and III there was no statistically significant differences (7).

Therefore, in total, it seems that administration of intravaginal progesterone to support the luteal phase in patients may be a better choice. Although the results in all outcomes expect abortion rate was not statistically significant. This may-be due to the low sample size in our study which is the main limitation of the present investigation. Our study, for the first time, has compared three different protocols for luteal phase support by progesterone administration. 

In conclusion, we suggest the use of intravaginal progesterone during the luteal phase in patients undergoing an IVF-ET program because low the numbers of abortions, high and ongoing pregnancy rates. However, further studies utilizing larger sample sizes are required in order to determine the best treatment for luteal phase support in patients who undergoing IVF treatment.
